# Development and validation of an oropharyngeal obstruction evaluation score

**DOI:** 10.1007/s11325-023-02978-y

**Published:** 2024-01-05

**Authors:** Bengang Peng, Mo Chen, Jing Yang, Youqing Lai, Ning Zhang

**Affiliations:** Department of Otorhinolaryngology, Beijing Jishuitan Hospital, Capital Medical University, 31 East Xinjiekou Street, Xicheng District, Beijing, 100035 China

**Keywords:** Sleep apnea, Obstructive sleep apnea prediction, Oropharyngeal obstruction evaluation score, Receiver operating characteristic curve analysis

## Abstract

**Objective:**

To identify standard clinical parameters that can predict the presence and severity of obstructive sleep apnea.

**Subjects and methods:**

Adult patients with habitual snoring completed comprehensive polysomnography and anthropometric measurements, including sex, age, body mass index (BMI), neck circumference, tonsil size grading, modified Mallampati score, and nasofibroscopy-assisted Muller’s maneuver (NMM). Spearman’s correlation coefficient was used to screen the significant variables. Stepwise multiple linear regression analysis was then conducted to identify the independent variables. receiver operating characteristic (ROC) curve analysis was used to quantify the predictability of the formed oropharyngeal obstruction scoring system.

**Results:**

A total of 163 adults (127 men) were enrolled in the study. Tonsil size grading, modified Mallampati score, and NMM grading maneuver were predictive of  OSA and incorporated into a scoring system. This score ranged between 3 and 12, and threshold values of ≥ 8 and ≥ 9 seemed to be appropriate to identify patients at an increased risk of at least mild (AHI ≥ 5/h; AUROC = 0.935, 95%CI = 0.900–0.970, *P* < 0.001) and severe OSA (AHI ≥ 30/h; AUROC = 0.939, 95%CI = 0.899–0.969, *P* < 0.001), respectively.

**Conclusion:**

This study established an evaluation score for assessing the degree of oropharhygeal obstruction. The findings of the study suggest that the score may help identify patients at risk of oropharyngeal-related OSA who should have a full sleep evaluation.

## Introduction

The prevalence of obstructive sleep apnea (OSA) has increased rapidly in recent years. Approximately, 10–17% of men and 3–9% of women suffer from OSA [1]. Common symptoms reported by patients, such as snoring, lethargy, nonrestorative sleep, and excessive daytime sleepiness, as well as systematic diseases like hypertension [2], coronary artery disease, and stroke [3, 4], are linked to untreated OSA.

Obstruction or collapse of the upper airway, which consists of nasopharyngeal, oropharyngeal, and laryngopharyngeal portions, plays a very important role in the development of OSA. Although surgery for patients with OSA is considered to be an important option, night time ventilation therapy which uses continuous positive-airway pressure (CPAP) remains the standard therapy [5]. Thus, evaluation of the upper airway obstruction of patients is important to develop their therapeutic strategy.

The oropharyngeal level is the most common obstruction site of OSA due to the lack of bony structural support. The soft palate, the base of tongue, the tonsil size, and the collapse of the oropharyngeal airway are places where potential obstruction occurs. Unfortunately, there is no unified scale or score to evaluate the obstruction of the oropharynx, leaving the evaluation to the clinician’s personal experience. Therefore, a comprehensive and objective score for evaluation of oropharyngeal obstruction would be useful.

The main airway assessments at the oropharyngeal level include tonsil size, Mallampati score, nasofibroscopy-assisted Muller’s maneuver (NMM), CT scan of the upper airway, and drug-induced sleep endoscopy [6]. However, all these assessments are either insufficient to make a treatment decision in OSA or are invasive and unfeasible in the outpatient setting. For example, tonsil size grading and Mallampati score for tongue position are the traditional methods used to evaluate the oropharyngeal obstruction, but they can only demonstrate the obstruction in a single dimension for awake patients. CT is a three-dimensional measurement and CT is therefore commonly used for volumetric and linear evaluation of the upper airway. A drawback of CT is that it exposes patients to radiation [7]. NMM is an endoscopic test that a patient can easily receive at clinic. It is performed while the patient is awake [8] and therefore, the morphology of the upper airway during sleep cannot be observed. Drug-induced sleep endoscopy (DISE) is now the most accuate assessment to evaluate the upper airway during sleep. However, DISE is invasive, time-consuming, and costly [9].

Therefore, the objective of this study was to develop a reliable score for evaluation of oropharyngeal obstruction to help identify patients  at-risk for OSA. The oropharyngeal obstruction score was first derived from a sleep clinic cohortand its prediction accuracy for OSA was then validated by ROC analysis.

## Materials and methods

### Subjects

This retrospective study was approved by the Institutional Review Board of the Beijing Jishuitan Hospital Ethical Committee. All methods were performed in accordance with the relevant guidelines and regulations by the Institutional Review Board of the Beijing Jishuitan Hospital Ethical Committee.

**Fig. 1:**
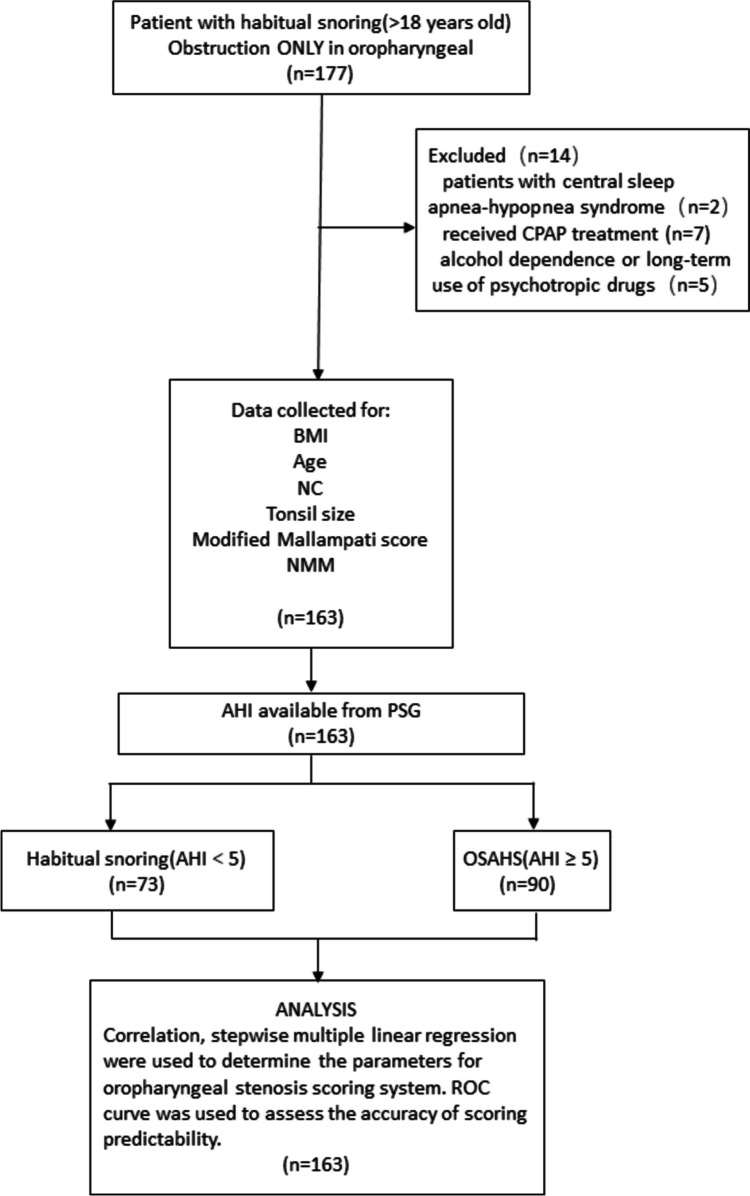
The flowchart illustrating the recruitment process and the study design

A retrospective analysis was performed on adult patients (> 18 years old) with habitual snoring and obstruction only in oropharyngeal level who were admitted to Beijing Jishuitan Hospital from June 2015 to June 2020. The medical history, age, gender, height, weight, and other basic information of the patients were recorded. Relevant physical examinations and routine examinations of otorhinolaryngology department were conducted. Patients with the following conditions were excluded from this study: (1) patients with severe heart, lung, liver, and kidney dysfunctions; (2) patients with sleep breathing disorders caused by special reasons such as thyroid dysfunction, hypopituitarism, acromegaly (gigantism), narcolepsy, myasthenia gravis, recurrent laryngeal nerve palsy, etc.; (3) patients with central sleep apnea; (4) patients with alcohol dependence or long-term use of psychotropic drugs; and (5) patients who had received previous OSA-related treatment, such as ventilator treatment, surgical treatment (such as palate pharyngoplasty, nasal dilatation, upper and lower jaw surgery), and oral appliance therapy. The flowchart of the screening process is shown in Fig. 1.

## Anthropometric and oropharyngeal airway anatomic variables

The following indices were considered in this research:

I. Body mass index (BMI, kg/m2) was calculated as the ratio of the weight (in kg) to the square of the height (in m).
II. Neck circumference (in cm) was measured just below the laryngeal prominence with the head positioned in the Frankfort horizontal plane.
III. Tonsil size grading was adopted in this study as: (1) tonsils were hidden within the pillars; (2) tonsils extended to the pillars; (3) tonsils were beyond the pillars but not to the midline; and (4) tonsils extended to the midline.
IV. The modified Mallampati score of each patient was assessed in a sitting position with maximal tongue protrusion (without using a tongue depressor). A scaled score was derived based on the anatomical ratio of tongue to the structures of the oropharyngeal: (1) tonsils, palatal arches, and soft palate were all visualized; (2) uvula and palatal arches were visualized; (3) only soft palate was visualized; and (4) only hard palate was visualized [10].
V. NMM was used to evaluate the collapse of the oropharyngeal airway. It was performed with patients in the supine position and the Frankfort plane perpendicular to horizontal. Prior to the procedure, patients were given instructions for the Muller maneuver and given time to practice until they were comfortable performing it. When performing the Muller maneuver, patients were asked to perform a maximal inspiration against a closed airway. Following the administration of topical anesthetic nasal spray (4% lidocaine), the nasopharyngoscope (Olympus Medical Systems Corp) was inserted through the anesthetized nasal cavity to the lower oropharynx. Images were taken during tidal breathing and the Muller maneuver. The position of the scope was kept still when obtaining images, to make consistent magnification among pictures. Visual monitoring during the procedure was required so that an orthogonal view was maintained and captured for subsequent analysis. The lateral pharyngeal walls’ (LPWs) collapse during the Muller Maneuver was assessed and classified into four levels: (1) slight (collapse < 25%); (2) moderate (collapse ≥ 25% but < 50%); (3) substantial (collapse ≥ 50% but < 75%); and (4) predominant (collapse ≥ 75%) [11].

### Polysomnogram (PSG) and diagnosis

PSG (CompuMedics, Australia) was completed in the ENT Department of Beijing Jishuitan Hospital. Electroencephalography, submental electromyography, and electro-oculography were recorded with surface electrodes by standard techniques. OSA was defined as a reduction in airflow by more than 90% of the baseline for at least 10 s with effort to breathe. Obstructive hypopnea was an abnormal respiratory event characterized by a reduction of at least 30% in thoracoabdominal movement or airflow, for at least 10 s and with more than 4% of blood oxygen saturation reduction, when compared to the baseline [12]. Apnea hypopnea index (AHI) was defined as the total number of the obstructive apneas and hypopneas within 1 h of sleep under electroencephalography. OSA was diagnosed when the AHI was higher than 5/h. All PSG findings were recorded by physicians.

### Statistical methods

All statistical analysis was conducted with SPSS 22.0 for Windows (I SPSS Inc., Chicago, IL). First, Spearman’s correlation coefficient was adopted to identify the relationships between the clinical variables and the AHI. Next, a stepwise multiple linear regression analysis was conducted for the significant variables selected in the first step, to distinguish the independent clinical variables. These variables were then grouped to the oropharyngeal stenosis scoring system. Receiver operating characteristic (ROC) curve analysis was conducted and the area under ROC curve (AUROC) was calculated to quantify the predictability of oropharyngeal stenosis scoring system. The statistical significance of the above analysis was set at *P* < 0.001.

## Results

Among 163 patients, 127 were men. Ages of the patients ranged from 18 to 68 years with a mean of 40.2 years. The average BMI of the patients was 27.2 kg/m^2^, with the standard deviation of 4.7 kg/m^2^. The clinical OSA predictors, i.e., anthropometry and oropharyngeal anatomy variables, and AHI values, are shown in Table 1. Seventy-three (45%) patients had simple habitual snoring with an AHI < 5/h. Seventeen patients (10%) who had AHI higher than 5/h but lower than 15/h were considered to have mild OSA; 21 patients (13%) with AHI of 15/h and up to 30/h had moderate OSA; and 52 (32%) had severe OSA with AHI ≥ 30/h.

**Table 1:** Baseline clinical data^a ^

Variables	Mean (SD)	Range
Age, y	40.2 (11.1)	18–68
Anthropometry and oropharyngeal plane anatomy		
BMI, kg/m^2^	27.2 (4.7)	20–54
NC, cm	41.8 (5.1)	31–60
Tonsil size grading	2.4 (1.0)	1–4
Modified Mallampati Score	2.4 (1.0)	1–4
NMM grading	2.4 (1.1)	1–4
Polysomnography, AHI/h	1.3 (1.3)	1–4

Abbreviations: *AHI*, apnea/hypopnea index; *BMI*, body mass index; *NC*, neck circumference. *NMM*, nasopharyngoscopy assistant Muller maneuver

^a^Patients, *n* = 163 (36 women, 127 men)

### Correlations between clinical OSA predictors and AHI values

The AHI scores differed between sex (*P* = 0.019; Table 2). Spearman’s correlation coefficients exhibited significant positive correlation between AHI values and multiple clinical predictors, including BMI, NC, tonsil size grading, modified Mallampati score, and NMM grading. Their Spearman’s coefficients are 0.419, 0.399, 0.652, 0.593, and 0.707, respectively.

**Table 2:** Correlation between AHI and clinical predictor variables

Variables (*N* = 163)	Correlation coefficient^a^	*P* value
Sex	− 0.180*	0.021
Age	0.070	0.372
BMI	0.419**	< 0.001
NC	0.399**	< 0.001
Tonsil size grading	0.652**	< 0.001
Modified Mallampati score	0.593**	< 0.001
NMM grading	0.707**	< 0.001

^**^Correlation is significant at the 0.01 level (two-tailed)

^*^Correlation is significant at the 0.05 level (two-tailed)

^a^Spearman correlation coefficient

### Stepwise multiple linear regression for the model

To find the appropriate parameters to compose the oropharyngeal stenosis scoring system, a stepwise multiple linear regression analysis was performed to determine the significant predictor variables of the regression model (Table 3). The stepwise multiple linear regression analysis excluded NC and sex from the final model. The AHI for patients with oropharyngeal obstruction was a function of four predictor variables, and was written as the form as follows:

**Table 3:** Stepwise multiple linear regression of AHI grading with predictor variables^a^

Variable	Coefficient		VIF	*P* value
	B	Std. error		
Intercept	− 2.620	0.376		< 0.001
Muller grading	0.407	0.075	1.745	< 0.001
Tonsil size grading	0.435	0.075	1.536	< 0.001
Modified Mallampati score	0.314	0.073	1.379	< 0.001

^a^Dependent variable, AHI grading; *VIF* variance inflation factor

*r*^2^ = 0.658

$$\mathrm{AHI}\hspace{0.17em}=\hspace{0.17em}0.407\hspace{0.17em}\times\hspace{0.17em}\mathrm{NMM}\;\mathrm{grading}\hspace{0.17em}+\hspace{0.17em}0.435\hspace{0.17em}\times\hspace{0.17em}\mathrm{tonsil}\;\mathrm{size}\;\mathrm{grading}\hspace{0.17em}+\hspace{0.17em}0.314\hspace{0.17em}\times\hspace{0.17em}\mathrm{modified}\;\mathrm{Mallampati}\;\mathrm{score}\hspace{0.17em}+\hspace{0.17em}0.043\hspace{0.17em}\times\hspace{0.17em}\mathrm{BMI}\hspace{0.17em}-\hspace{0.17em}2.620$$

From the equation, the coefficients of NMM grading, tonsil size grading, and modified Mallampati score were similar (ranged from 0.314 to 0.435), and the coefficient of BMI was much smaller than the other three variables. It indicated that BMI was much less important in determining the AHI, while NMM grading, tonsil size grading, and modified Mallampati score were of almost equal importance. After checking the variance inflation factor of each predictor variable against one another, no substantial indication of multicollinearity was found. Therefore, these variables were treated as potential independent predictors.

### Establishment of the Peng score

In this study, we defined the oropharyngeal stenosis score system consisting of NMM grading + tonsil size grading + modified Mallampati score, and named it as Peng score. We set four points for each part based on the similar coefficients in the regression model, with full score of 12 points. Scoring criteria are listed in Table 4.

**Table 4:** Scoring system for oropharyngeal plane stenosis consists

Predictors		Scores
NMM grading		
	0 ~ 25% (excluding 25%)	1
	25 ~ 50% (excluding 50%);	2
	50 to 75% (excluding 75%)	3
	≥ 75%	4
Tonsil size grading		
	No enlargement of the tonsils	1
	Tonsil degree I, tonsil swelling over the palatoglossal arch, not over the palatopharyngeal arch	2
	Tonsil degree II, tonsil swelling beyond the palatoglossal arch, but not reaching the midline of the posterior pharyngeal wall	3
	Tonsil degree III, tonsil enlargement reaching or exceeding the midline of the posterior pharyngeal wall	4
Modified Mallampati score		
	Grade I, can see the soft palate, uvula, tonsil, palatopharyngeal arch	1
	Grade II, with soft palate, partial uvula,, and palatopharyngeal arch visible	2
	Grade III, only the base of the soft uvula can be seen	3
	Grade IV, with only hard palate visible	4
Peng score		Sum of above

### Validation of the Peng score

**Fig. 2:**
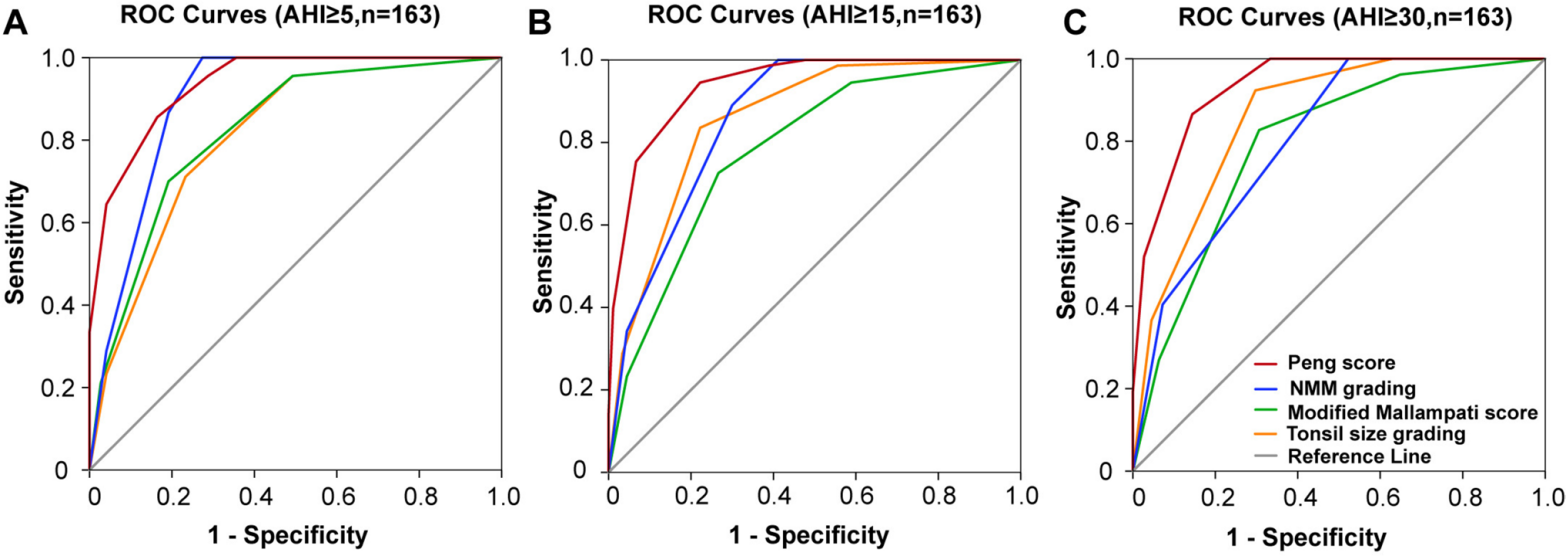
Illustrations of ROC curves for the scores (orange lines tonsil size grading, green lines modified Mallampati score, blue lines NMM grading, red lines Peng score). Results concern the ability of the scores to detect at least mild (**A**), moderate to severe (**B**), and severe (**C**) OSA

The sensitivity and specificity of the Peng score, along with tonsil size grading, modified Mallampati score, and NMM grading, were used to plot receiver operating characteristic (ROC) curves (shown in Fig. 2) of the three following groups: the presence of OSA (AHI ≥ 5/h), the presence of moderate/severe OSA (AHI ≥ 15/h), and the presence of severe OSA (AHI ≥ 30/h). Accuracy of each grading system was measured by the area under the ROC (AUROC) curve.

The value larger than 0.9 often represents extremely good discrimination. Table 5 lists the statistics observed from ROC curve for AHI ≥ 5, including AUROC, sensitivity, specificity, corresponding value for diagnosis, and standard error. The corresponding value of 7.5 for Peng score (sensitivity = 0.836, specificity = 0.692) may be useful to predict the presence of OSA (AHI ≥ 5/h; AUROC = 0.935, 95%CI = 0.900–0.970, *P* < 0.001). The same corresponding value for Peng score (sensitivity = 0.945, specificity = 0.778, shown in Table 6) may also be useful to predict the presence of moderate OSA (AHI ≥ 15/h; AUROC = 0.939, 95%CI = 0.904–0.973, *P* < 0.001). An increased cutoff value of 8.5 for Peng score (sensitivity = 0.865 specificity = 0.856) was obtained with ROC analysis to predict the present of severe OSA (AHI ≥ 30/h; AUROC = 0.939, 95%CI = 0.899–0.969, *P* < 0.001; shown in Table 7).

**Table 5:** Statistics observed from ROC curve for AHI ≥ 5(Fig. 2A)

Test result variable(s)	Area under the curve	Std. error^a^	Asymptotic sig.^b^	Asymptotic 95% confidence interval		YI max	Corresponding value	Sensitivity	Specificity
				Lower bound	Upper bound				
Tonsil size grading	0.808	0.035	< 0.001	0.740	0.876	0.478	2.5	0.711	0.767
Modified Mallampati score	0.823	0.033	< 0.001	0.757	0.888	0.508	2.5	0.7	0.808
NMM grading	0.896	0.028	< 0.001	0.841	0.950	0.675	2.5	0.867	0.808
Peng score	0.935	0.018	< 0.001	0.900	0.970	0.856	7.5	0.836	0.692

**Table 6:** Statistics observed from ROC curve for AHI ≥ 15 (Fig. 2B)

Test result variable(s)	Area under the curve	Std. error^a^	Asymptotic sig.^b^	Asymptotic 95% confidence interval		YI max	Corresponding value	Sensitivity	Specificity
				Lower bound	Upper bound				
Tonsil size grading	0.856	0.029	< 0.001	0.799	0.913	0.614	2.5	0.836	0.778
Modified Mallampati score	0.781	0.036	< 0.001	0.710	0.851	0.459	2.5	0.726	0.733
NMM grading	0.859	0.028	< 0.001	0.803	0.915	0.59	2.5	0.89	0.7
Peng score	0.939	0.017	< 0.001	0.904	0.973	0.723	7.5	0.945	0.778

**Table 7:** Statistics observed from ROC curve for AHI ≥ 30 (Fig. 2C)

Test result variable(s)	Area under the curve	Std. error^a^	Asymptotic sig.^b^	Asymptotic 95% confidence interval		YI max	Corresponding value	Sensitivity	Specificity
				Lower bound	Upper bound				
Tonsil size grading	0.861	0.028	< 0.001	0.805	0.916	0.626	2.5	0.923	0.703
Modified Mallampati score	0.793	0.036	< 0.001	0.721	0.864	0.521	2.5	0.827	0.694
NMM grading	0.807	0.033	< 0.001	0.742	0.872	0.477	1.5	1	0.477
Peng score	0.934	0.018	< 0.001	0.899	0.969	0.721	8.5	0.865	0.856

## Discussion

In this study, we derived an evaluation score, the Peng score, for assessing the degree of oropharyngeal airway obstruction. This score ranges between 3 and 12, and threshold values of ≥ 8 and ≥ 9 appear to be appropriate to identify patients at an increased risk of at least mild and severe OSA, respectively. From the data obtained in this study, the sensitivity and specificity of Peng score were 83.6% and 69.2% for detecting patient with mild OSA (AHI ≥ 5) and 86.5% and 85.6% for patient with severe OSA (AHI ≥ 30), respectively. Compared with previous OSA-related scores, the Peng score has the potential to offer a low cost and easily accessible strategy in comprehensive and consistent evaluation of oropharyngeal obstruction, and it has the potential to play an important role in future diagnostic and treatment decisions.

Our proposed score has three main advantages: First, the score is based on three parameters which include tonsil size, tongue position, and NMM, assessing the coronal diameters, sagittal diameters, and the collapse of the oropharyngeal airway, respectively. 

Second, three variables of the score can be easily implemented during the examination in the outpatient setting and within a short time. The information regarding tonsil size and modified Mallampati score can be gathered by a clinician during routine physical exam. Nasofibroscopy, which is used during NMM, regularly available in an ENT clinic. All these assessments can be safely finished within a few minutes.

Compared with current scores, the proposed score exhibits greater predictive accuracy (AUROC = 0.935). The STOP-Bang Questionnaire [13, 14] and OSA50 [15], which consider snoring, tiredness, observed apnea, high blood pressure, body mass index (BMI), age, neck circumference (NC), and male sex, have the OSA prediction accuracy of 0.626 and 0.712, respectively [16]. The P-SAP [17], OSA-score [18], and DES-OSA [19], which include morphometry parameters like tongue position and tonsil size, have OSAS prediction accuracy range from 0.739 to 0.809 [16, 18]. Kljajić found strong positive association of AHI with modified Mallampati score, as well as positive correlation of AHI with tonsillar size in the multivariate forward stepwise regression analysis in children (range from 2 to 9 years old) [20]. A derived model based on the local clinical findings (modified Mallampati score, tonsil size, adenoid size, age, gender, and body mass index) significantly overlapped with the results of an overnight polysomnography in diagnosing OSA in children. The sensitivity of the tested model was 84%, and specificity was 74% [21]. There are two possible reasons for the high OSA predictive accuracy of the newly proposed score: (1) The score is specifically designed for the evaluation of oropharyngeal obstruction and prediction of risk for oropharyngeal-related OSA, while other scores are applicable for patients with different kinds of OSA. As mentioned before, obstructions in any level of upper airway can lead to OSA, which complicates the ability to sort out the cause and degree of obstruction. (2) NMM mimics the collapse of oropharyngeal airway under obstruction and the proposed scoring systsem is the only system that includes NMM which increases its predictive accuracy.

The current study has limitations: (1) The inter-observer variability of Muller maneuver and MS is a limitation. To minimize inter-observer variability as much as possible, we have employed a strategy of standardized training for our observers prior to data collection. (2) The newly proposed score cannot distinguish moderate OSA (AHI ≥ 15) from mild OSA (AHI ≥ 5) because a threshold value of > 7.5 was chosen for both groups (shown in Table 5 and 6). (3) This study was performed in a sleep center, where the subjects have higher likelihood of OSA than the subjects in the general population; i.e., the distribution of subjects in this study may not reflect the actual distribution in general population. These limitations may serve as sources of bias in our results. 

In conclusion, this study describes a new scoring system to evaluate the obstruction of the oropharyngeal airway, based on morphologic characteristics and NMM. The findings suggest that thenew score may be effective in identifying patients at risk of oropharyngeal OSA. Future studies with larger sample sizes and patients with different degrees of OSA severity may be required to validate the model’s predictive ability.

## Data Availability

All data generated or analyzed during this study are included in this article. Further enquiries can be directed to the corresponding author.
